# Choroid plexus volumes in patients with transient global amnesia: A retrospective study

**DOI:** 10.1097/MD.0000000000040077

**Published:** 2024-10-11

**Authors:** Dong Ah Lee, Ho-Joon Lee, Geunyeol Jo, Kang Min Park

**Affiliations:** a Department of Neurology, Haeundae Paik Hospital, Inje University College of Medicine, Busan, Republic of Korea; b Department of Radiology, Haeundae Paik Hospital, Inje University College of Medicine, Busan, Republic of Korea; c Department of Rehabilitation Medicine, Haeundae Paik Hospital, Inje University College of Medicine, Busan, Republic of Korea.

**Keywords:** choroid plexus, glymphatic system, transient global amnesia

## Abstract

Increased choroid plexus (ChP) volume is well known to be associated with glymphatic system dysfunction. This study aimed to investigate glymphatic system function in patients with transient global amnesia (TGA) compared to healthy controls through ChP volumes measurements. We retrospectively enrolled patients with TGA from our hospital, as well as healthy controls. This was retrospectively observational study followed STROBE guideline. All participants underwent brain magnetic resonance imaging, including three-dimensional T1-weighted imaging. We analyzed and compared ChP volumes between patients with TGA and healthy controls and investigated the relationship between ChP volumes and clinical characteristics in patients with TGA. We enrolled 44 patients with TGA and 47 healthy controls. Among the 44 patients with TGA, 38 experienced a single TGA event, while 6 had recurrent TGA events. ChP volumes did not significantly differ between patients with TGA and healthy controls (2.140% vs 2.089%, *P* = .568). However, ChP volumes were higher in patients with a single TGA event compared to those with recurrent events (2.204% vs 1.740%, *P* < .013). We observed a significant positive correlation between ChP volumes and age in patients with TGA (*R* = 0.282, *P* = .007). ChP volumes were not associated with the duration of amnesia in patients with TGA (*R* = 0.187, *P* = .274). We find no differences in the glymphatic system function, as demonstrated by ChP volume for the first time. This study also found a significant correlation between ChP volume and age in patients with TGA, indicating that aging influences glymphatic system function.

## 1. Introduction

Transient global amnesia (TGA), a neurological disorder, is characterized by an acute, self-limiting episode of anterograde amnesia accompanied by retrograde amnesia of variable extent.^[1,2]^ The typical clinical presentation involves a sudden inability to retain entirely new information, lasting for several hours, with resolution typically occurring within 24 hours.^[1,3]^ TGA predominantly affects middle-aged or elderly people.^[1]^ Alternative causes that could precipitate amnesia must be excluded, and TGA is diagnosed based on the Hodges–Warlow criteria.^[4]^

The pathophysiology of TGA remains unclear. Numerous studies have proposed several explanatory mechanisms, including ischemic processes, epileptic seizures, cortical spreading depression observed in migraines, and venous insufficiency.^[2]^ Although the etiology of TGA remains incompletely understood, reports estimate the recurrence rate of TGA to be between approximately 2% to 27%. Several clinical characteristics of patients, including female sex, migraine history, depression, hypothyroidism, atrial fibrillation, prior head injury, or a family history of dementia, are reportedly the associated factors, as evidenced by multiple investigations.^[5–7]^ In a previous study, we demonstrated the feasibility of using deep learning to diagnose TGA based on electroencephalography (EEG), distinguishing between patients with recurrent TGA events and those with a single TGA event.^[8]^ Additionally, we previously reported on the differences in brain networks between patients with recurrent TGA events and those with a single TGA event by analyzing the functional brain network using cerebral blood flow measurements from arterial spin labeling magnetic resonance imaging (MRI).^[9]^

The choroid plexus (ChP) is located within each ventricle of the brain and serves as a secretory tissue accountable to produce cerebrospinal fluid (CSF). It consists of a monolayer of polarized secretory epithelial cells, which represents a continuation of the ependymal lining of the ventricles. This distinctive structural characteristic not only contributes to CSF production but also facilitates the formation of the blood–cerebrospinal fluid barrier.^[10,11]^ ChP plays a crucial role in maintaining the homeostasis of the central nervous system, including normal brain growth, neurogenesis, influence of hormones, and involvement in circadian thyrhms.^[12]^ It regulates the expression of receptors for different proteins and pathogens and is rich in the expression of various immunological cells.^[13,14]^

Recent research on ChP has focused on its connection to the glymphatic system, which is the waste clearance system of the central nervous system.^[15–17]^ The glymphatic system facilitates CSF influx from the subarachnoid space through periarterial spaces into the brain parenchyma, where it mixes with interstitial fluid (ISF). Several factors can influence ISF through bulk flow, including CSF inflow, arterial pulsatility, hydrostatic pressure differences between arterial and venous perivascular spaces, and osmotic gradients. Studies in animals demonstrate that ISF and its solutes migrate towards the venous perivascular space, where the fluid is absorbed and removed from the brain parenchyma through convection.^[18–20]^ Recent research findings suggest that ChP volume may be associated with glymphatic system dysfunction.^[15,21]^ Consequently, numerous studies have been published on the volume of ChP in various neurological disorders, particularly neurodegenerative diseases.^[17,22,23]^

This study aimed to compare ChP volume between patients with TGA and healthy controls. Additionally, we investigated whether ChP volume differed according to the recurrence of TGA events. This study intended to examine the association between ChP volume and glymphatic system function, thereby elucidating the contribution of glymphatic system function to the pathophysiology of TGA.

## 2. Methods

### 2.1. Participants

The study was conducted in compliance with the Declaration of Helsinki and was approved by the Institutional Review Board of our hospital.

This retrospective case–control study was conducted at a single tertiary hospital. Certified neurologists diagnosed TGA according to Hodges and Warlow criteria.^[4]^ Exclusion criteria included the presence of structural lesions on brain MRI, except for hippocampal dot lesions on diffusion-weighted images, and the detection of epileptiform discharges on EEG. All participants underwent three-dimensional T1-weighted MRI imaging (3D-T1WI), and clinical data were collected from patients with TGA through chart review. Additionally, we included healthy controls who were age- and sex-matched and had no prior history of medical or neurological disease. Individuals with identified structural lesions on their brain MRI were excluded.

### 2.2. MRI acquisition

Both patients with TGA and healthy controls underwent brain MRI using a 3-tesla MRI scanner with a 32-channel head coil (Achieva Tx, Philips Healthcare, Best, The Netherlands). 3D-T1WI was obtained using the turbo-field echo sequence, with the following parameters: inversion time = 1300 ms, repetition time/echo time = 8.6/3.96 ms, flip angle = 8°, and voxel size = 1 mm³ isotropic. The same imaging sequences were acquired for both groups.

### 2.3. Choroid plexus volume analysis

**Figure 1.**
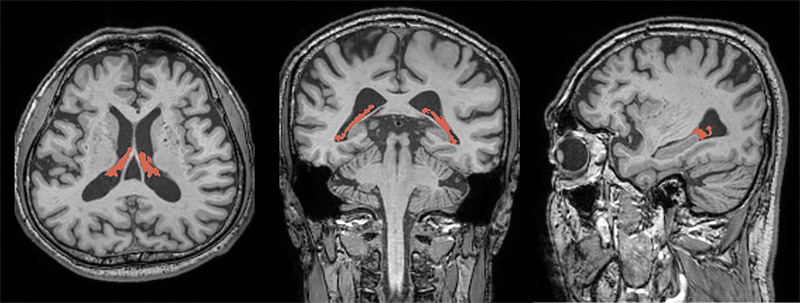
Choroid plexus segmentation. Representative images showing choroid plexus segmentation (red) overlaid on three-dimensional T1-weighted magnetic resonance images in the axial (left), coronal (center), and sagittal (right) planes.

We utilized the same method used in our previous research for the volume analysis of ChP.^[24]^ Briefly, ChP was automatically segmented using Gaussian mixture model-based segmentation.^[25]^ 3D-T1WI was corrected for bias field using the sequence-adaptive multimodal segmentation pipeline.^[26]^ Subsequently, the volumes of the right and left lateral ventricle masks and the segmentation-based total intracranial volume were acquired using SynthSeg with the bias-corrected images as input.^[27]^ The Gaussian mixture model was then applied to the bias-corrected 3D-T1WI to separate ChP from the CSF and ventricular walls as distinct clusters of voxel intensities within the lateral ventricle masks. A board-certified neuroradiologist with 10 years of experience examined and further refined ChP masks resulting from the automated pipeline to remove any obvious non-ChP areas (septum pellucidum, ventricular walls, flow artifacts, or noise within the CSF, etc). The volumes of the final masks were calculated and normalized using the segmentation-based total intracranial volume (Fig. 1).

### 2.4. Statistical analyses

Clinical characteristics including age, sex, and ChP volumes were compared between patients with TGA and healthy controls using the independent *t* test or Chi-squared test. Pearson correlation analysis was used to analyze the relationship between ChP volume and clinical characteristics. All statistical analyses were conducted using MedCalc® Statistical Software version 22.016 (MedCalc Software Ltd., Ostend, Belgium; https://www.medcalc.org; 2023). Statistical significance was set at *P* < .05 for all calculations.

## 3. Results

### 3.1. Clinical characteristics of the study population

Table 1 shows the demographic and clinical characteristics of patients with TGA and healthy controls. We enrolled 44 patients with TGA and 47 healthy controls. Of the 44 patients with TGA, 38 had a single TGA event, whereas 6 had recurrent TGA events. The demographic and clinical characteristics such as age, sex, EEG abnormality, hippocampal dot lesion on diffusion weighted image, duration of amnesia, precipitating factors, and past medical history between the patients with a single TGA event and those with recurrent TGA events were not different.

**Table 1:** The demographic and clinical characteristics of patients with TGA and healthy controls.

	Patients with TGA (N = 44)	Healthy controls (N = 47)	*P*-value
Age, mean years (±SD)	61.1 ± 8.3	60.4 ± 7.7	.634
Male, n (%)	16 (36.4)	17 (36.2)	.985

	Patients with a single TGA event (N = 38)	Patients with recurrent TGA events (N = 6)	*P*-value
Age, mean years (±SD)	61.3 ± 7.6	60.2 ± 12.4	.750
Male, n (%)	13 (34.2)	3 (50.0)	.460
EEG abnormalities, n (%)	6 (15.8)	1 (16.7)	.957
Hippocampal dot lesion on DWI, n (%)	16 (42.1)	3 (50.0)	.720
Duration of amnesia, median hours (IQR)	5 (2.5–10.0)	5 (3.5–9.0)	.761
Precipitation factor, n (%)	17 (44.7)	3 (50.0)	.812
Emotional stress, n	10	1	
Physical activity, n	4	2	
Temperature change, n	4	0	
Past medical history, n (%)	11 (28.9)	2 (33.3)	.829
Hypertension, n	6	1	
Dyslipidemia, n	3	2	
Diabetes, n	2	0	
Others, n	4	0	

DWI = diffusion-weighted imaging, EEG = electroencephalography, IQR = interquartile range, SD = standard deviation, TGA = transient global amnesia.

### 3.2. Choroid plexus volume analysis

**Figure 2.**
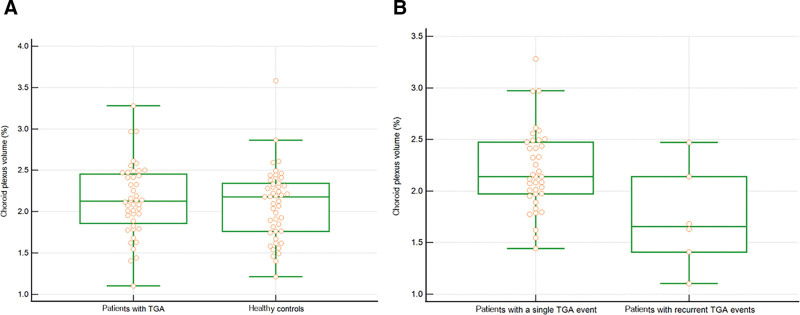
Comparison of choroid plexus volume between patients with TGA and healthy controls. Box and whisker plots show no significant difference in ChP volumes between patients with TGA and healthy controls (2.140% vs 2.089%, *P* = .568) (A). The choroid plexus volumes in patients with a single TGA event were higher than those in patients with recurrent TGA events (2.204% vs 1.740%, *P* < .013) (B). ChP = choroid plexus, TGA = transient global amnesia.

ChP volumes did not significantly differ between patients with TGA and healthy controls (2.140% vs 2.089%, *P* = .568) (Fig. 2A). However, ChP volumes in patients with a single TGA event were higher than those in patients with recurrent TGA events (2.204% vs 1.740%, *P* < .013) (Fig. 2B).

### 3.3. Correlation between choroid plexus volume and clinical characteristics

**Figure 3.**
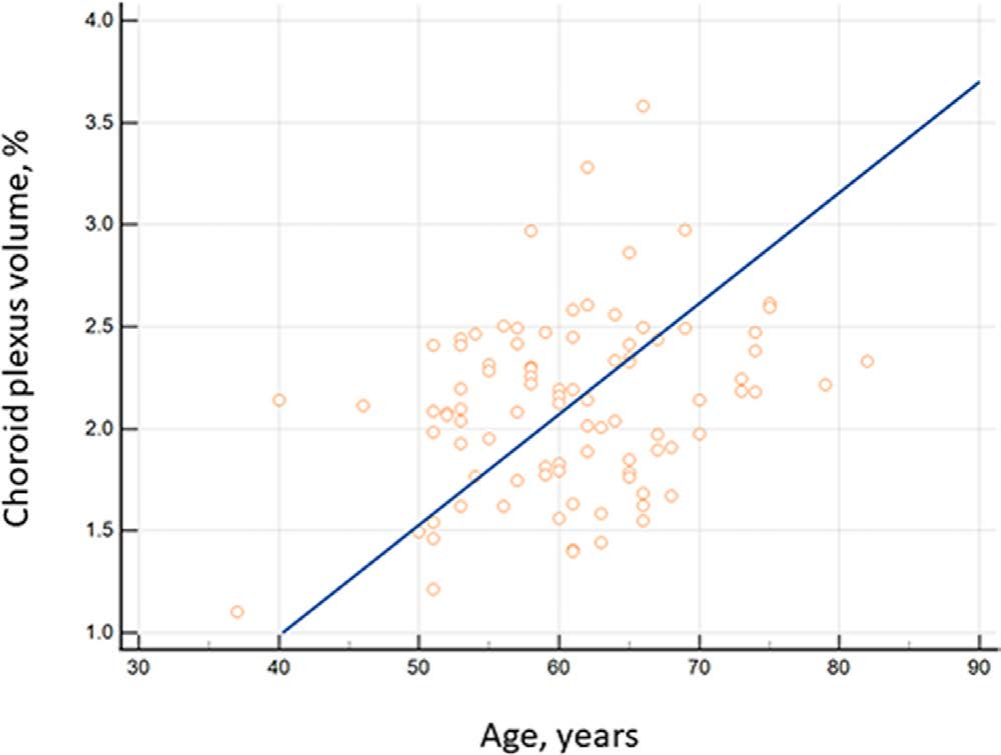
Correlation between choroid plexus volumes and clinical characteristics in patients with TGA. The figure shows that choroid plexus volume is positively correlated with age (*R* = 0.282, *P* = .007). TGA = transient global amnesia.

We observed a significant correlation between ChP volume and age of participants (*R* = 0.282, *P* = .007) (Fig. 3).

ChP volumes were not significantly associated with the duration of amnesia in patients with TGA (*R* = 0.187, *P* = .274).

## 4. Discussion

This study aimed to evaluate glymphatic system function using ChP volume in patients with TGA. ChP volumes did not differ significantly between patients with TGA and healthy controls. However, patients with a single TGA event had higher ChP volumes than those with recurrent TGA events. A significant correlation was observed between ChP volume and age, but not between ChP volume and the duration of amnesia.

The lack of significant difference in ChP volume between the patients with TGA and healthy controls suggests that glymphatic system function is comparable between these groups. This finding is consistent with our previous study, which aimed to evaluate glymphatic system function in patients with TGA using the diffusion tensor image (DTI) analysis along the perivascular space method.^[28]^ This may be associated with the fact that patients with TGA did not exhibit other neurological symptoms apart from amnesia and that TGA itself follows a benign natural course. Consequently, brain MRI performed during or immediately after the amnesia event would likely show no differences compared to healthy controls, except for the presence of hippocampal dot lesions on diffusion weighted image. In a systematic review of TGA’s long-term prognosis, long-term risks of dementia, epilepsy, psychological-emotional disturbances, vascular events, and mortality were evaluated. While no differences in the long-term risks of vascular events, mortality, and psychological disturbances were found between patients with TGA and healthy controls, conflicting results were reported for dementia and epilepsy across different studies.^[29]^ In the study by Hsieh et al, the cumulative incidence of dementia over an 8-year period following a TGA episode was reported to be higher in the TGA group.^[30]^ In contrast, Arena et al reported no difference in the 12-year cumulative risk of dementia between the groups.^[31]^ Furthermore, Hsieh et al reported that the 8-year risk of epilepsy was higher in patients with TGA compared to healthy controls,^[32]^ whereas Arena et al found no difference in epilepsy risk between the 2 groups.^[31]^ If we were to investigate whether a TGA episode affected the glymphatic system function in patients, it would have been necessary to conduct follow-up brain MRI to evaluate ChP volume after the single TGA event. However, due to our retrospective study design, we did not have access to brain MRI scans taken at regular intervals over a specific period. Additionally, analysis would have been feasible with sufficient patients with recurrent TGA events.

We found that ChP volumes in patients with a single TGA event were higher than those in patients with recurrent TGA events (2.204% vs 1.740%, *P* < .013). However, interpreting this as indicating more severe glymphatic dysfunction in the single event group compared to the recurrent events group is difficult. This could be explained as a type 1 error due to an imbalance in the number of subjects in both groups. Further studies with large sample size are needed.

The glymphatic system is affected by aging due to a decrease in CSF influx into the glymphatic pathway and a reduction in clearance efficacy.^[18,19,33]^ In animal model studies, amyloid-β clearance reportedly decreases by approximately 40% in old age, leading to the discovery that advancing age is associated with a dramatic decline in the efficiency of exchange between the subarachnoid CSF and the brain parenchyma.^[34]^ Also, a 27% reduction in the vessel wall pulsatility of intracortical arterioles and widespread loss of perivascular aquaporin-4 protein (AQP4) polarization along the penetrating arteries have been demonstrated.^[34,35]^ In postmortem analysis of elderly cognitively intact individuals and patients with Alzheimer disease, the expression of AQP4 was associated with advancing age across all individuals. Additionally, perivascular AQP4 localization significantly correlated with Alzheimer disease status, independent of age, and the loss of perivascular AQP4 localization was associated with increased amyloid-β burden.^[36]^ In this study, we confirmed that the volume of ChP positively correlates with age. An increase in ChP volume is an indicator of glymphatic system dysfunction.^[15,21]^ In a study of idiopathic normal pressure hydrocephalus, gadobutrol is reportedly absorbed from cerebrospinal fluid by the ChP. Additionally, delayed clearance in the idiopathic normal pressure hydrocephalus group compared to healthy controls confirms the direct involvement of the ChP in the glymphatic pathway.^[21]^ In a study utilizing 2 cohorts, the first cohort underwent glymphatic MRI 39 hours after intrathecal administration of contrast, while the second cohort evaluated glymphatic function indirectly using the DTI-analysis along the perivascular space index based on DTI in participants from the CIRCLE study (ClinicalTrials.gov: NCT03542734). In both cohorts, higher ChP volume was associated with glymphatic system dysfunction.^[15]^ In recent research, ChP enlargement is particularly associated with clinical characteristics in neurodegenerative diseases, and is consistently correlated with cognitive decline.^[17,22,23,37]^ In particular, in neurodegenerative diseases, a significant correlation between disease progression and an increase in ChP volume has been reported.^[22,23,37]^

This study is the first to compare the volume of ChP between patients with TGA and healthy controls to evaluate differences in glymphatic system function. However, this study has some limitations. First, the sample size was relatively small, and the study was conducted at a single center. Second, all patients with TGA underwent MRI during the ictal or peri-ictal phase of amnesia; however, follow-up MRI comparisons were not performed. Due to the cross-sectional study design, changes in glymphatic system function in patients who have experienced an amnestic event were not tracked, even though most cases of TGA have a benign natural course as a single event. This underscores the necessity for additional research in the future.

We find no differences in glymphatic system function, as demonstrated by ChP volume for the first time. This study also reveals that the significant correlation between ChP volume and age in patients with TGA indicates that glymphatic system function is influenced by aging.

## Author contributions

**Conceptualization:** Kang Min Park.

**Data curation:** Geunyeol Jo.

**Formal analysis:** Kang Min Park.

**Methodology:** Kang Min Park.

**Project administration:** Kang Min Park.

**Software:** Ho-Joon Lee.

**Validation:** Dong Ah Lee, Ho-Joon Lee, Kang Min Park.

**Visualization:** Dong Ah Lee, Kang Min Park.

**Writing – original draft:** Dong Ah Lee, Ho-Joon Lee, Geunyeol Jo, Kang Min Park.

**Writing – review & editing:** Kang Min Park.
